# Completing the Enalaprilat Excretion Pathway—Renal Handling by the Proximal Tubule

**DOI:** 10.3390/pharmaceutics12100935

**Published:** 2020-09-30

**Authors:** Nori J. L. Smeets, Carlijn H. C. Litjens, Jeroen J. M. W. van den Heuvel, Hedwig van Hove, Petra van den Broek, Frans G. M. Russel, Jan B. Koenderink, Saskia N. de Wildt

**Affiliations:** 1 Department of Pharmacology and Toxicology, Radboud Institute of Health Sciences, Radboud University Medical Center, 6525 EZ Nijmegen, The Netherlands; nori.smeets@radboudumc.nl (N.J.L.S.); carlijn.litjens@radboudumc.nl (C.H.C.L.); jeroen.vandenheuvel@radboudumc.nl (J.J.M.W.v.d.H.); hedwig.vanhove@radboudumc.nl (H.v.H.); petra.vandenbroek@radboudumc.nl (P.v.d.B.); frans.russel@radboudumc.nl (F.G.M.R.); jan.koenderink@radboudumc.nl (J.B.K.); 2 Department of Pharmacy, Radboud Institute for Health Sciences, Center for Infectious Diseases, Radboud University Medical Center, 6525 EZ Nijmegen, The Netherlands; 3 Department of Intensive Care and Pediatric Surgery, Erasmus MC—Sophia Children’s Hospital, 3015 GJ Rotterdam, The Netherlands

**Keywords:** enalapril, enalaprilat, drug transporters, proximal tubule cell

## Abstract

Background: Enalapril is often used in the treatment of cardiovascular diseases. Clinical data suggest that the urinary excretion of enalaprilat, the active metabolite of enalapril, is mediated by renal transporters. We aimed to identify enalaprilat specificity for renal proximal tubular transporters. Methods: Baculovirus-transduced HEK293 cells overexpressing proximal tubular transporters were used to study enalaprilat cellular uptake. Uptake into cells overexpressing the basolateral transporters OCT2, OAT1, OAT2, or OAT3 and apical transporters OAT4, PEPT1, PEPT2, OCTN1, OCTN2, MATE1, MATE2k, and URAT1 was compared with mock-transduced control cells. Transport by renal efflux transporters MRP2, MPR4, P-gp, and BCRP was tested using a vesicular assay. Enalaprilat concentrations were measured using LC-MS/MS. Results: Uptake of enalaprilat into cells expressing OAT3 as well as OAT4 was significantly higher compared to control cells. The enalaprilat affinity for OAT3 was 640 (95% CI: 520–770) µM. For OAT4, no reliable affinity constant could be determined using concentrations up to 3 mM. No transport was observed for other transporters. Conclusion: The affinity of enalaprilat for OAT3 and OAT4 was notably low compared to other substrates. Taking this affinity and clinically relevant plasma concentrations of enalaprilat and other OAT3 substrates into account, we believe that drug–drug interactions on a transporter level do not have a therapeutic consequence and will not require dose adjustments of enalaprilat itself or other OAT3 substrates.

## 1. Introduction

With an increase in both the prevalence and incidence of cardiovascular disease over the past 25 years [1], the number of patients who require treatment is rising. Treatment often comprises complex regimens in which angiotensin-converting-enzyme (ACE) inhibitors are frequently included [2]. Enalapril is an ACE inhibitor used for the treatment of hypertension, heart failure, and chronic kidney diseases (CKD) and, although it is a commonly prescribed drug, the effect of enalapril therapy shows significant interindividual variability that is not completely understood [3].

**Figure 1:**
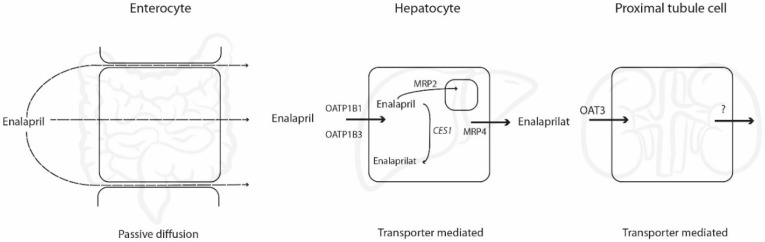
Processes governing absorption, metabolism, and excretion of enalapril and enalaprilat in the intestine, liver, and kidney. CES1: carboxylesterase 1.

Various physiological processes, including drug metabolism as well as active uptake and efflux by different transporters, govern the systemic exposure of enalapril and its active metabolite enalaprilat. Knowledge about these processes helps to explain variability in effect as well as to better understand possible drug–drug interactions. Enalapril is used to treat cardiovascular disease and therefore often used in combination therapy. In theory, this increases the risk of drug–drug interactions and, therefore, the risk of altered efficacy or toxicity, both of enalapril itself and/or the other drug(s). Many of these processes governing enalapril’s disposition have been identified and are shown in Figure 1.

Uptake of enalapril in the small intestine occurs via passive diffusion [4], followed by the entrance of enalapril in the hepatocyte mediated by the organic anion transporting polypeptides (OATP) 1B1 and 1B3 [5]. In the hepatocyte, enalapril is metabolized via carboxylesterase 1 (CES1) to its active metabolite enalaprilat [6]. Although enalapril is considered a specific CES1 substrate, conversion of approximately 32% to enalaprilat by CES2 has also been reported in freshly prepared rat intestinal homogenate, but no human data are available [7]. Following its formation, enalaprilat is transported into the systemic circulation via multidrug resistance-associated protein 4 (MRP4) [8]. A minority of enalapril remains untransformed and is excreted into bile via multidrug resistance-associated protein 2 (MRP2) [5]. Renal elimination of enalaprilat is thought to occur through both glomerular filtration as well as tubular secretion (i.e., active transport), as enalaprilat clearance exceeds the clearance of the glomerular filtration rate marker inulin [9].

**Figure 2:**
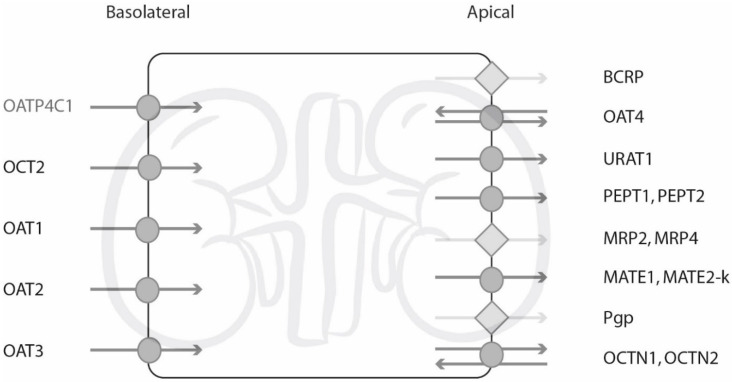
Human renal uptake transporters in the basolateral and apical membrane. In this study, transporters depicted as a circle in darker grey were investigated using a cellular assay, and diamond-shaped transporters in light grey with a vesicular assay. Figure adapted from Giacomini et al., Ivanyuk et al., and Yin et al. (references in text).

The kidney proximal tubule plays a pivotal role in the excretion of both endogenous and exogenous compounds and many transporters reside in the basolateral and apical membrane of the proximal tubule cell (Figure 2).

Understandably, changes in the tubular secretion of drugs may alter their systemic concentrations and thereby their efficacy and/or toxicity. As 50% of enalaprilat is bound to plasma proteins, only half of the reported clearance of 120 mL/min can be attributed to GFR (Glomerular Filtration Rate), emphasizing the importance of tubular secretion in the excretion of the remaining 50% [10,11]. This transporter-mediated secretion is assumed to be facilitated by an organic anion transporter (OAT), as enalaprilat is a weak acid [12] and its excretion is significantly reduced in vivo in the presence of probenecid, a competitive inhibitor of organic anion transporters [10]. It was recently described by Ni et al. that the secretion of enalaprilat by the basolateral proximal tubule cells is mediated by organic anion transporter 3 (OAT3). Based on in vitro experiments, suggestions for possible drug–drug and drug–herb interactions were made [13]. However, since clinically important interactions with enalapril have not been reported before, it raises questions about the in vivo relevance of these findings. Furthermore, the transporters at the apical side of the proximal tubule responsible for the elimination of enalaprilat into primary urine remain unknown. Here, we aimed to identify the membrane transporters involved in the renal proximal tubule transport of enalaprilat in order to explain the interindividual variability in its pharmacokinetics and to understand the near absence of clinically relevant drug–drug interactions.

## 2. Material and Methods

We used human embryonic kidney 293 (HEK293) cells overexpressing transporters known to be located in the proximal renal tubule to assess in vitro whether a substrate is also an in vivo transporter substrate [14]. Both a cellular and vesicular assay were used to assess the renal transporter specificity for enalaprilat. Methods were adapted from. Te Brake et al. (2016) [15].

Materials. Enalaprilat was purchased from Sanbio (Uden, The Netherlands). Model substrates were used to confirm uptake capacity of each transporter. [^3^H]-Glycyl-sarcosine (PEPT1/2), [^14^C]-Carnitine (OCTN1/2), and [^14^C]-Metformin (MATE1/2k and OCT2) were purchased from Moravek Biochemicals (Brea, CA, USA), while 5(6)-Carboxy-2′,7′-dichlorofluorescein (CDCF) (OAT1 and MRP2) and Urate (URAT1) were purchased at Sigma-Aldrich (St. Louis, MO, USA). [^14^C]-para-aminohippuric acid (OAT2), [^3^H]-Estrone sulfate (OAT3 and BCRP), and [^3^H]-Dehydro-epiandrosterone sulfate (MRP4) were purchased from PerkinElmer (Waltham, MA, USA), and [^3^H]-NMQ from Solvo Biotechnology (Szeged, Hungary) was used to confirm transport activity of P-gp. D_5_-enalaprilat (Toronto Research Chemicals, North York, Ontario, Canada) was used as an internal standard for liquid chromatography–tandem mass spectrometry (LC-MS/MS) quantification.

Hanks’ balanced salt solution (HBSS) and GlutaMAX high-glucose Dulbecco’s modified Eagle medium (DMEM) were obtained by Life Technologies Invitrogen (Carlsbad, CA, USA). Fetal bovine serum (FBS) was purchased from Greiner Bio-One (Sollingen, Germany) and bovine serum albumin (BSA) was obtained from Roche Diagnostics GmbH (Mannheim, Germany). Sodium butyrate and HEPES both were purchased from Sigma-Aldrich. BD BioCoat poly–d-lysine-coated 24-well tissue culture plates were ordered from Corning (New York, NY, USA). High-quality (UPLC/MS)-grade methanol was purchased from Boom (Meppel, The Netherlands) and NaOH from Merck (Darmstadt, Germany). Protein concentrations were determined with a colorimetric assay kit (Bio-Rad Protein Assay) from Bio-Rad Laboratories (Hercules, CA, USA).

The recombinant baculoviruses that contain the cDNA of human transporters OCT2, OAT1, OAT2, OAT3, OAT4, BCRP, URAT1, PEPT1, PEPT2, MRP2, MRP4, MATE1, MATE2-k, URAT1, BCRP, P-gp, OCTN1, and OCTN2 downstream of a cytomegalovirus (CMV) promotor were generated by our department. Generation of these vectors took place as previously described [16]. Amino acid sequences were the same as those of GenBank accession no. NM_153276.2 for OAT1, NM_006672 for OAT2, NM_004254.3 for OAT3, NM_018484.3 for OAT4, NM_003058.4 for OCT2, NM_003059.2 for OCTN1, NM_003060.3 for OCTN2, NM_018242.3 for MATE1, NM_001099646.2 for MATE2k, NM_144585.3 for URAT1, NM_005073.3 for PEPT1, NM_021082.3 for PEPT2, and NM_004827.2 for BCRP.

Cell line maintenance and transduction: HEK293 cells were maintained at 37 °C with 5% CO_2_ in DMEM containing 10% FBS. After growing to 70% confluence, ~300,000 cells were seeded in poly-d-lysine-coated 24-well plates in a volume of 400 µL per well. Twenty-four hours after seeding, cells were transduced with 60 µL recombinant baculovirus to express the transporter of interest. As transporter specificity was tested in transiently transduced cells, control cells underwent the same transduction procedure with enhanced yellow fluorescent protein (EYFP) as control (background). To enhance protein expression, 140 µL DMEM containing sodium butyrate (3 mM final concentration) was added to each well. Uptake experiments were performed 3 days post transduction.

Identification of enalaprilat transporters: Prior to testing enalaprilat specificity, transporter functionality was checked using model substrates. For OAT1 and OAT3, this was previously described by El-Sheikh et al. [17]. For MRP2, MRP4, P-gp, and BCRP, this was described by Lempers et al. [18]. MATE1, MATE2k, and OCT2 specificity were tested by Van der Velden et al. [19] (Table 1). OAT2 and OAT4 were tested using [^14^C]-PAH, PEPT1 and PEPT2 using [^3^H]-Gly-Sar, URAT1 using urate, and OCTN1 and OCTN2 using [^14^C]-Carnitine (Supplementary Figure S1). On the day of the experiment, the culture medium was removed and cells were first washed in order to equilibrate them to serum-free conditions with 400 µL buffer (HBSS plus 10 mM HEPES, pH 7.4), prewarmed to 37 °C. Uptake was initiated by replacing this solution with 150 µL of fresh transport buffer (HBSS-HEPES, pH 7.4, except for MATE2k (pH 8.5) and PEPT1 (pH 6), kept at 37 °C) supplemented with the 10 µM enalaprilat solution incubated at 37 °C for 10 min.

For practical reasons, MATE1 and MATE2k activity levels were measured in the uptake direction due to the nature of the cellular assay, even though, in the kidney and liver, the MATEs export organic cations into the urine and bile, respectively. Furthermore, also OAT4 activity was measured in the uptake direction, whereas this transporter is a bidirectional transporter in the kidney. To terminate the reaction, transport buffer was replaced with 400 µL ice-cold HBSS-HEPES buffer containing 0.5% BSA (*m*/*v*), after which the cells were washed twice with ice-cold HBSS-HEPES. To measure the intracellular enalaprilat concentrations, cells were solubilized in 200 µL 50% methanol. Aliquots were analyzed using LC-MS/MS.

If cells transfected with a transporter of interest showed statistically significant uptake of enalaprilat compared to control cells, inhibition of this transport was tested to prove the role of this specific transporter. This was the case for OAT3 and OAT4. MK571 was used to demonstrate inhibition of enalaprilat transport as MK571 is an established multidrug resistance protein inhibitor and was recently demonstrated to be a high-affinity inhibitor for OAT1, OAT2, and OAT3 [20]. To enable comparison between inhibitory experiments, OAT4 was also inhibited using MK571, although the inhibitory potential for OAT4 has not been described before. Enalaprilat (10 µM) uptake experiments were conducted in the presence and absence of a MK571 (10 µM) solution and cells were incubated for 20 min. When significant uptake and inhibition were observed, further experiments were conducted to determine time-dependent and concentration-dependent transport for the selected transporters. Time-dependent uptake of enalaprilat (10 µM) was assessed by performing transport assays at various incubation times, namely 5, 10, 20, 30, 45, and 60 min. Concentration-dependent transport was determined by performing uptake assays at various concentration ranges (1, 3, 10, 30, 100, 300, 1000, and 3000 µM) after 20 min of incubation at 37 °C. Finally, to determine the inhibitory potency of enalaprilat on OAT3 transport, 20 nM [^3^H]-E1S was used as an OAT3 model substrate with increasing enalaprilat concentrations up to 3000 µM. This experiment was performed in triplicate.

Protein concentration: In each transporter assay, the average amount of cellular protein per transporter transduction was determined to be able to display the results in a standardized way (uptake in pmol/mg protein/minute). Cells were solubilized in 200 µL 1 M NaOH and protein concentrations were determined using the Bio-Rad protein assay kit with BSA as a standard.

Specificity for MRP2, MRP4, P-gp, and BCRP. In order to determine enalaprilat specificity for the efflux transporters MRP2, MRP4, P-gp, and BCRP, a vesicular assay was used. As no transport was observed but enalaprilat specificity for MRP4 has been previously described [8], experiments were repeated, applying double transduction in a cellular assay.

*Vesicular assay:* Uptake of enalaprilat into membrane vesicles was performed as described previously [21]. In brief, membrane vesicles (7.5 µg) were prewarmed for 1 min at 37 °C and added to PharmTox assay buffer (PharmTox, Nijmegen, The Netherlands) supplemented with 4 mM ATP or AMP (control) and 10 mM MgCl_2_ in a final volume of 30 µL. The reaction mixture was incubated at 37 °C for 10 min. Subsequently, samples were transferred to a 0.45 µm MultiScreen_HTS_ HV filter plate (PVDF, Merck) by using a Multiscreen™ Vacuum Manifold 96-well filtration device (Millipore, Bedford, MA, USA) and washed twice with PharmTox stop-wash buffer. Vesicles were lysed with 100 µL 25% ACN and aliquots were analyzed using liquid LC-MS/MS as described below.

*Cellular double transduction assay:* Cells were cultured and transduced as described above; however, since enalaprilat is hydrophilic, an uptake transporter (OAT3) had to be incorporated together with the efflux transport of interest to measure specificity for these efflux transporters in a cellular assay. Therefore, HEK293 cells were co-transduced with 30 µL recombinant baculovirus expressing OAT3 and 60 µL recombinant baculovirus coding for either MRP2, MRP4, or P-gp. This enables the determination of a significant reduction in uptake due to the presence of an efflux transporter. This method of co-transduction was previously described by van der Velden et al. as a valid method to identify efflux transporter specificity. In their article, they demonstrated a statistically significant decrease in the uptake of the OCT1 substrate proguanil in cells co-transduced with OCT1 and MATE-2k, compared to OCT1 alone [19]. Quantification of enalaprilat: Enalaprilat was quantified with LC-MS/MS, using an Acquity UPLC (Waters, Milford, MA, USA) coupled to a Xevo TQ-S (Waters) triple quadropole mass spectrometer. Separation took place using a BEH C18 analytical column (1.7 μm; 50 × 2.1 mm, Acquity UPLC^®^, Waters, Ireland). As an internal standard, d_5_-enalaprilat was used. The following elution gradient was used: 0–1 min, from 20% B to 80% B; 1–2 min, 100% B; and 2–3 min, 20% B. Solvent A consisted of 0.1% formic acid in H_2_O and solvent B consisted of 0.1% formic acid in methanol. The column temperature was set at 40 °C, and the flow rate was 300 µL/min. The effluent from the UPLC was passed directly into the electrospray ion source. Positive electrospray ionization was achieved using nitrogen as a desolvation gas with ionization voltage at 1000 Volt. The source temperature was set at 500 °C and argon was used as collision gas. Detection of enalaprilat and the internal standard was based on isolation of the protonated molecular ion, [M + H]^+^, and subsequent MS/MS fragmentations and a multi reaction monitoring (MRM) were carried out. The following MRM transitions were used: for enalaprilat, *m*/*z* 349.1 (parent ion) to *m*/*z* 206.1 and 91.0 (both product ions), and for d5-enalaprilat, *m*/*z* 354.2 (parent ion) to *m*/*z* 211.1 and 95.9 (both product ions).

Analysis. Data were expressed as means ± standard error of the means (SEM) from three independent experiments. Statistically significant differences in uptake (*p* < 0.05) were assessed using one-way analysis of variance (ANOVA) followed by Dunnett’s post hoc multiple-comparison test in the case of the initial transporter screen. Mean uptake ratios of substrate specific transport were calculated by dividing specific transport by control (background transport). To determine statistically significant differences in uptake between incubation with and without the presence of an inhibitor, a paired T-test was applied. All statistical analyses were performed using SPSS version 25 (IBM Corp., Armonk, NY, USA). To estimate maximum transport velocity (V_max_) and the substrate concentration at which half of this rate is obtained (K_m_), concentration-dependent uptake data were fitted to the Michaelis–Menten equation and calculated using GraphPad Prism Version 5.3 (GraphPad Software Inc, San Diego, CA, USA). As V_max_ values are dependent on the transporter expression in the particular system used, transport velocity was depicted as percentage of the V_max_. Non-linear regression analysis was used to calculate IC_50_ values with GraphPad Prism, with a bottom = 0 constraint. Assuming competitive inhibition, the inhibition constant (K_i_) value was extrapolated from the IC50-values according to the Cheng–Prusoff equation. Due to the low substrate concentration, the IC_50_ is equal to the K_i_.

## 3. Results

By using both a cellular and vesicular assay, we were able to assess the renal transporter specificity for enalaprilat.

**Figure 3:**
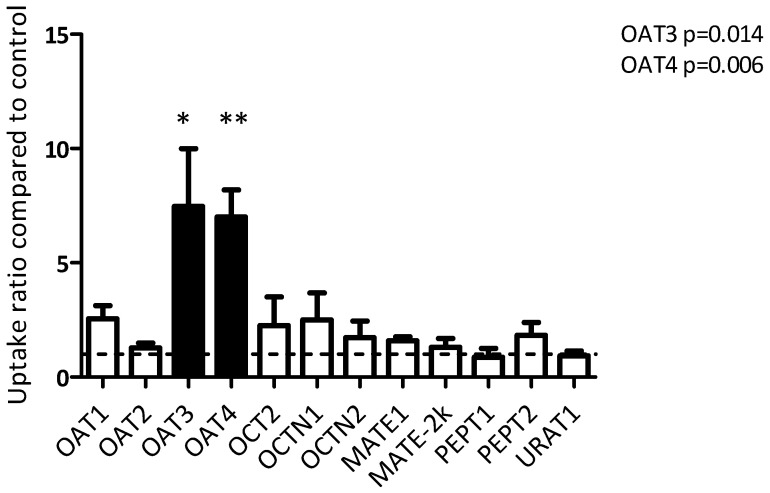
Uptake of enalaprilat was mediated by OAT3 and OAT4 in HEK293 cells transiently overexpressing a single uptake transporter. Transport is expressed as ratio of control cells and presented as means ± SEM. Data were pooled from three independent experiments. Dotted line represents a ratio between transporter and control of 1 (i.e., no active transport). An asterisk indicates a statistically significant difference from the control by one-way ANOVA using Dunnett’s post hoc test (* *p* < 0.05, ** *p* < 0.01).

Screening of uptake transporters. HEK293 cells transiently overexpressing one of the uptake transporters present in the kidney proximal tubule cells [14,22,23] were used to determine the transporter specificity for enalaprilat. All relevant transporters showed uptake of their model substrate. For OAT1, OAT3, OCT2, MATE1, MATE2k, MRP2, MRP4, P-gp, and BCRP, this has been previously described [15,17,18,19]. OAT2, OAT4, PEPT1, PEPT2, URAT1, OCTN1, and OCTN2 all showed a significant transport ratio compared to control cells and were thus also considered suitable for testing enalaprilat specificity (Supplementary Figure S1). Of all tested transporters, only OAT3 (*p* = 0.014) and OAT4 (*p* = 0.006) mediated statistically significant uptake of enalaprilat compared to control cells (Figure 3).

**Figure 4:**
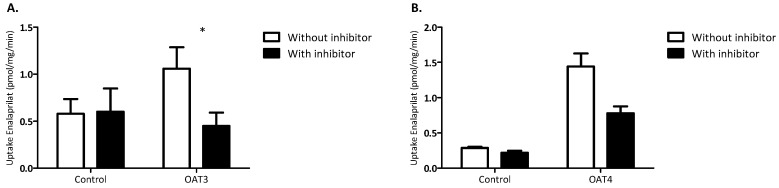
Transport of enalapril by OAT3 and OAT4 was effectively inhibited by the OAT inhibitor MK571. Effect of the OAT inhibitor MK571 (10 µM) on the uptake of enalaprilat into HEK293 cells overexpressing OAT3 (**A**) and OAT4 (**B**). Transport is expressed as pmol/mg/min and presented as mean ± SEM. Data were pooled from three independent experiments. An asterisk indicates a statistically significant difference from the non-inhibited transporter by a paired sample *T*-test (* *p* < 0.05).

Inhibition of OAT3- and OAT4-mediated enalaprilat uptake. To confirm specific transport of enalaprilat by OAT3 and OAT4, uptake experiments were performed with or without the OAT inhibitor MK571. As shown in Figure 4, uptake of enalaprilat by OAT3 was statistically significantly reduced in the presence of MK571 (*p* < 0.05). Uptake of enalaprilat by OAT4 was visually reduced in the presence of MK571, though this difference was not significant (*p* = 0.07).

Time-dependent uptake of enalaprilat. In order to determine the optimal incubation time, enalaprilat uptake was tested at 5, 10, 20, 30, 45, and 60 min. Uptake (1 µM) by OAT3 was linear for up to 30 min (results not shown), and for OAT4, a similar trend was observed. As accurate concentration dependency can only be determined within the linear phase of transport, further experiments were performed using an incubation time of 20 min for both OAT3 and OAT4.

**Figure 5:**
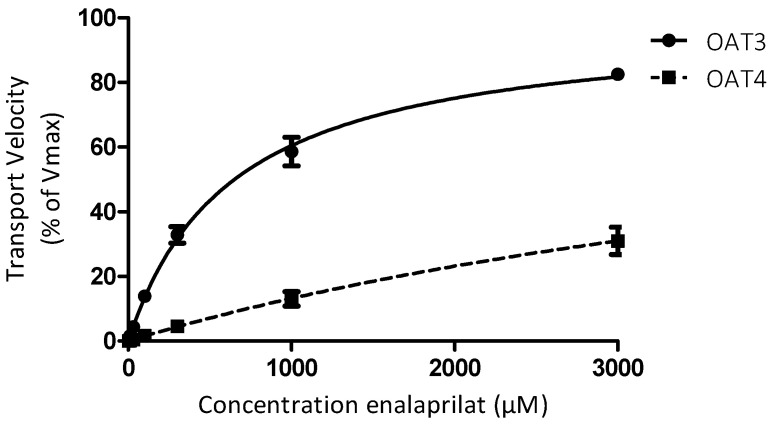
Concentration-dependent uptake of enalaprilat by OAT3 and OAT4. Michaelis–Menten kinetic curves showing the relationship between enalaprilat concentration and transport velocity of OAT3 and OAT4. For OAT3, a Km value of 640 µM (95% CI 510–770 µM) was observed, whereas saturation of transport via OAT4 was not reached over the concentration range measured. Transport velocity is expressed in percentage of maximum velocity and presented as mean ± SEM for each data point. Data were pooled from three independent experiments.

Concentration-dependent uptake of enalaprilat. Figure 5 shows the Michaelis–Menten curves of enalaprilat for uptake via OAT3 and OAT4. The apparent affinity constant of enalaprilat for OAT3 was 640 µM (95% confidence interval [CI] 510–770 µM), and for OAT4, it was clearly higher than 1000 µM but could not be reliably determined using concentrations up to 3 mM.

Inhibitory potency of enalaprilat against OAT3. Transport of [^3^H]-E1S by OAT3 was determined in the presence of increasing enalaprilat concentrations. Exposure to enalaprilat concentration-dependently inhibited the uptake of ^3^H-E1S with a K_i_ value of 134 µM (Supplementary Figure S2).

Specificity for MRP2, MRP4, P-gp, and BCRP. For the efflux transporters MRP2, MRP4, BCRP, and P-gp, of which MRP4 has been described previously as relevant for hepatic efflux of enalaprilat into the systemic circulation, we tested the specificity of these transporters for enalaprilat in order to understand apical transport [14]. Using both a vesicular assay as well as a double-transduction approach in cells, enalaprilat transport by MRP2, MRP4, BCRP, or P-gp did not differ from control cells or vesicles (Supplementary Figure S3).

## 4. Discussion

In this study, we assessed the in vitro affinity of enalaprilat for clinically relevant proximal tubule transporters and showed that transport of enalaprilat is mediated by OAT3 and OAT4. We also demonstrated a clear decrease in uptake in the presence of the OAT inhibitor MK571, which confirms our finding that OAT3 and OAT4 are responsible for basolateral uptake and apical efflux of enalaprilat. To the best of our knowledge, we are the first to report transporter specificity for 16 renal tubular transporters.

We were able to reliably determine the apparent affinity constant for the uptake transporter OAT3, whereas this value could not be calculated for OAT4 because saturation of transport did not show any sign of saturation up to a concentration of 3 mM. A K_m_ of 640 µM demonstrates a relatively low affinity as compared to other OAT3 substrates, including many cardiovascular drugs that have a much higher affinity for OAT3 (i.e., furosemide 21.5 µM [24] and rosuvastatin 7.4 µM [25]).

The affinity constant that we report is in the same range as that of Ni et al. (640 vs. 284 µM). When taking enalapril concomitantly with other OAT3 interacting drugs, suppression of enalaprilat uptake by the proximal tubule could theoretically be expected, leading to higher serum concentrations. However, whether this is also relevant for the disposition of enalaprilat depends on both the K_I_ and the serum concentration of the drug. To further investigate the inhibitory potential of enalaprilat, the inhibitory potential of enalaprilat on other OAT3 substrates was attested, showing a K_i_ value of 134 µM.

Although many possible drug–drug and drug–herb interactions are suggested by Ni et al., we believe that the low affinity and relatively high IC_50_ explain the absence of studies in patients showing an increase in enalaprilat (i.e., as the victim) plasma concentrations or a relevant interaction with drugs taken concomitantly with enalapril (i.e., as the perpetrator drug). For example, the IC_50_ of the high-affinity OAT3 substrate furosemide is 7.3 µM [26] and observed therapeutic plasma concentrations are around 3.6 µM (total concentration) [27]. After co-administration of furosemide with enalapril at therapeutic dosages of both drugs (enalapril 10 mg and furosemide 80 mg), only a non-significant increase in both the enalaprilat C_max_ (increase from 34.9 to 38.2 ng/mL) and area under the curve (AUC) (increase from 369 to 384 ng/mL/h) were observed [28].

However, at high serum concentrations of an OAT3 substrate drug with a strong inhibitory potency, significantly reduced excretion of enalaprilat is seen. This was demonstrated by the effect of probenecid pretreatment (1000 mg once daily for 5 days) on enalaprilat PK in healthy volunteers. [10]. Probenecid is a strong OAT3 inhibitor with an IC_50_ of 4.4 µM [29]. Mean peak enalaprilat serum concentrations increased statistically significantly after probenecid pretreatment from 62 to 84 ng/mL, AUC increased by 50%, and mean clearance decreased from 110 to 66 mL/min after a single 20 mg enalapril dose. Not only pharmacokinetic (PK) parameters were affected—the pharmacodynamics of enalaprilat were also influenced by probenecid pretreatment as fractional excretions of sodium, calcium, magnesium, and urate were enhanced. Moreover, the effect of enalaprilat on the standing mean arterial pressure (MAP) was further enhanced by probenecid pretreatment (95 ± 2 to 83 ± 2 mmHg, *p* < 0.01). This suggests possible clinically relevant interactions between enalaprilat and organic anions with a high affinity for OAT3 as well as at (very) high plasma concentrations.

When taking the observed enalaprilat C_max_ and the affinity for OAT3 into account, we believe that clinically relevant concentrations of enalaprilat do not lead to saturation of OAT3. Moreover, the risk of influencing the disposition of other OAT3 substrates at the renal tubular level is extremely small with an IC_50_ of 135 µM. In heart failure patients, enalapril doses between 2.5 and 10 mg lead to a C_max_ of enalaprilat of approximately 40–110 ng/mL [30]. This corresponds to values of 0.11–0.32 µM, which is 2000-fold lower than the K_m_ (640 µM) and also significantly lower than the IC_50_ value (135 µM). Whether the disposition of enalaprilat changes due to the presence of other OAT3 substrates depends on their plasma concentration and inhibitory potency.

The K_m_ of OAT4 could not be determined, but based on our experiments, it is at least higher than 1 mM. Little is known about the role of OAT4 in the excretion of exogenous and endogenous substances. OAT4 is expressed in the apical membrane of the proximal tubule cell, where it operates as an asymmetric urate transporter, and it has been shown that the transport of substrates by OAT4 is bidirectional [14]. It is responsible for the secretion of organic anions into the lumen and it forms an entry point for endogenous substances like urate and estrone sulfate into the proximal tubule cell [31]. Since enalaprilat is an organic anion and OAT3 is responsible for its uptake from plasma across the basolateral membrane into the proximal tubular cells, we postulate that OAT4 is responsible for facilitating the apical efflux of enalaprilat into the primary urine.

Although it was described before that enalaprilat is excreted across the hepatic basolateral membrane by MRP4, we were not able to replicate this finding using two different experimental designs of which the vesicular transport assay was broadly similar to that described in the paper by Ferslew et al. [8]. However, minor differences in cell culture and membrane vesicle preparation protocols were observed. Ferslew et al. used a stably transfected MRP4 HEK293 cell line and used a different membrane isolation and transport assay protocol. These differences, however, do not explain the different results obtained. In addition, the transport activity observed was rather low and an affinity was not reported. Moreover, the hepatic expression of MRP4 is questionable. Drozdzik et al. could not detect MRP4 protein using LC-MS/MS and reported very low mRNA expression in hepatic tissue derived from deceased organ donors [32].

## 5. Limitations

In conclusion, the current study was designed to gain insight into the renal handling of enalaprilat. By knowing which transporter is responsible for the renal elimination of enalaprilat and its transporter kinetics, the absence of drug–drug interactions with frequently used cardiovascular drugs could be explained. In contrast, though age-related [33] and disease-associated [34] changes were described to alter transporter expression and pharmacokinetics of drugs, we do not believe that the large interindividual variability in enalaprilat exposure observed can be explained by variability in OAT3 and/or OAT4 activity. Taking the relatively low affinity of OAT3 and OAT4 into account, the probability of these factors to influence the disposition of enalaprilat is negligible as plasma concentration will stay far below the Km value at therapeutic dosages.

Based on our data, we postulate that OAT3 is responsible for the uptake at the basolateral membrane of the proximal tubules, whereas OAT4 transports enalaprilat into the primary urine. Because of the low affinity of enalaprilat for both transporters, we do not consider enalaprilat able to influence the disposition of other OAT3/4 substrates and vice versa. These data can possibly serve as input for physiologically based pharmacokinetic (PBPK) modeling to improve predictions about plasma concentrations of enalaprilat in different populations.

## Figures and Tables

**Table 1:** Previously described transporter activity for listed transporters. Fold change for cellular assay calculated by transport observed in cells transduced with transporter of interest divided by transport observed in cells transduced with EYFP. Fold change for vesicular assay calculated as transport observed in vesicles with ATP divided by those with AMP.

Transporter	Model Substrate (Assay)	Transport, Approx. Fold of Control (*p*-Value)	Inhibition of Transport Shown (Inhibitor)	Reference
OAT1	[^3^H]-MTX (cells)	3 fold (*p* < 0.05)	Yes (probenecid)	El Sheikh et al., 2013 [17]
OAT3	[^3^H]-MTX (cells)	3 fold (*p* < 0.05)	Yes (probenecid)	El Sheikh et al., 2013 [17]
OCT2	Cycloguanil (cells)	12 fold (*p* < 0.03)	Yes (multiple, inhibition of ASP most significant for Quinine)	Van der Velden et al., 2017 [19]
MATE1	Cycloguanil (cells)	4 fold (*p* < 0.01)	-	Van der Velden et al., 2017 [19]
MATE2K	Cycloguanil (cells)	5 fold (*p* < 0.001)	-	Van der Velden et al., 2017 [19]
MRP2	[^3^H]-MTX (vesicles)	4 fold	-	Lempers et al., 2016 [18]
MRP4	[^3^H]- E_2_17βG (vesicles)	5 fold	-	Lempers et al., 2016 [18]
*P*-GP	[^3^H]-NMQ (vesicles)	5 fold	-	Lempers et al., 2016 [18]
BCRP	[^3^H]-E1S (vesicles)	11 fold	-	Lempers et al., 2016 [18]

## Supplementary Materials

The following are available online at https://www.mdpi.com/1999-4923/12/10/935/s1, Figure S1: Transporters OAT2, OCTN1, OCTN2, PEPT1, PEPT2 and URAT1 show significant uptake of their model substrate, Figure S2: Concentration response curve for the inhibition of [3H]-E1S uptake by enalaprilat. Figure S3: Efflux transporters MRP2, MRP4, P-gp and BCRP did not show transport of enalaprilat.

## Abbreviations

[^3^H]-E1S	[^3^H]-estrone-sulfate ammonium salt
[^3^H]-MTX	[^3^H]-methotrexate
[^3^H]-NMQ	[^3^H]-*N*-methyl quinidine
ACE	angiotensin-converting-enzyme
AUC	area under the curve
ANOVA	analysis of variance
BSA	bovine serum albumin
BCRP	breast cancer resistance protein
CDCF	carboxy-2′,7′-dichlorofluorescein
CES1	carboxylesterase 1
CI	confidence interval
CKD	chronic kidney diseases
C_max_	maximum serum concentration
CMV	cytomegalovirus
DMEM	Dulbecco’s modified Eagle medium
E_2_17βG	estradiol 17β-d-glucuronide
EYFP	enhanced yellow fluorescent protein
FBS	fetal bovine serum
HBSS	Hanks’ balanced salt solution
HEK293	human embryonic kidney 293
HPLC	high-performance liquid chromatography
IC_50_	half maximal inhibitory concentration
K_m_	substrate concentration at which half of this speed is obtained
LC-MS/MS	liquid chromatography-tandem mass spectrometry
MATE1	multidrug and toxin extrusion 1
MATE2k	multidrug and toxin extrusion 2k
MRP2	multidrug resistance-associated protein 2
MRP4	multidrug resistance-associated protein 4
OAT	organic anion transporter
OAT1	organic anion transporter 1
OAT3	organic anion transporter 3
OATP	organic anion transporting polypeptides
OCT2	organic cation transporter 2
OCTN1	organic cation/carnitine transporter 1
OCTN2	organic cation/carnitine transporter 2
PEPT1	peptide transporter 1
PEPT2	peptide transporter 2
P-gp	P-glycoprotein
SEM	standard error of the mean
URAT1	urate transporter 1
V_max_	maximum transport velocity
