# Supplementary material for: Completing the Enalaprilat Excretion Pathway—Renal Handling by the Proximal Tubule
Source: Pharmaceutics. 2020 Sep 30;12(10):935. doi: 10.3390/pharmaceutics12100935 (PMC7600309; doi:10.3390/pharmaceutics12100935)
Supplement: Supplementary file 1 [file pharmaceutics-12-00935-s001.zip › Supplement_rebuttal/Supplementary files.docx]

Supplementary Files

**Figure S1.** Transporters OAT2, OCTN1, OCTN2, PEPT1, PEPT2, and URAT1 show significant uptake of their model substrate.

**Supplementary Figure S2**. Concentration response curve for the inhibition of [3H]-E1S uptake by S2

IC_50_ of enalaprilat was estimated at 135 µM. Uptake is depicted as mean ± SD for each data point. Experiment was performed in triplicate.

**Supplementary Figure S3.** Efflux transporters MRP2, MRP4, P-gp, and BCRP did not show transport of enalaprilat. (**A**) Transport of enalaprilat in membrane vesicles expressing MRP2, MRP4, P-gp, or BCRP in the presence of AMP (black bars) or ATP (checked bars). (**B**) Transport of enalaprilat in transiently transduced HEK293 cells in different combinations of control, the uptake transporter OAT3, and the efflux transporters MRP4 or P-gp. Transport is expressed in pmol/mg/min and presented as mean ± SEM for each data point. Data were pooled from three independent experiments.
